# Author Correction: HOIL-1L deficiency induces cell cycle alteration which causes immaturity of skeletal muscle and cardiomyocytes

**DOI:** 10.1038/s41598-024-61046-x

**Published:** 2024-05-06

**Authors:** Kentaro Akagi, Shiro Baba, Hiroaki Fujita, Yasuhiro Fuseya, Daisuke Yoshinaga, Hirohito Kubota, Eitaro Kume, Fumiaki Fukumura, Koichi Matsuda, Takayuki Tanaka, Takuya Hirata, Megumu K. Saito, Kazuhiro Iwai, Junko Takita

**Affiliations:** 1 Department of Pediatrics, Graduate School of Medicine, Kyoto University, 54 Kawahara-cho, Shogoin, Sakyo-ku, Kyoto City, Kyoto 606-8507 Japan; 2 Department of Molecular and Cellular Physiology, Graduate School of Medicine, Kyoto University, Yoshidakonoe-cho, Sakyo-ku, Kyoto City, Kyoto 606-8501 Japan; 3 Department of Clinical Application, Center for iPS Cell Research and Application (CiRA), Kyoto University, 53 Kawahara-cho, Shogoin, Sakyo-ku, Kyoto City, Kyoto 606-8507 Japan

Correction to: *Scientific Reports* 10.1038/s41598-024-57504-1, published online 17 April 2024

The Funding section in the original version of this Article contained an error. The Funding section now reads:

This work was supported by the Fujiwara Memorial Foundation (to KA (2020)), the Takeda Science Foundation (to TT (2017)), Grants-in-Aid for Scientific Research (to SB (20K08421) and SB (23K07529)), the Core Center for Regenerative Medicine and Cell and Gene Therapy (JP23bm1323001) from the Japan Agency for Medical Research and Development (AMED) (to M.K.S), and a grant from the iPS Cell Research Fund (to M.K.S).

The original Article has been corrected.

