# Supplementary material for: HOIL-1L deficiency induces cell cycle alteration which causes immaturity of skeletal muscle and cardiomyocytes
Source: Sci Rep. 2024 Apr 17;14:8871. doi: 10.1038/s41598-024-57504-1 (PMC11024103; doi:10.1038/s41598-024-57504-1)
Supplement: Supplementary file 1 — Supplementary Figures. [file 41598_2024_57504_MOESM1_ESM.pdf]

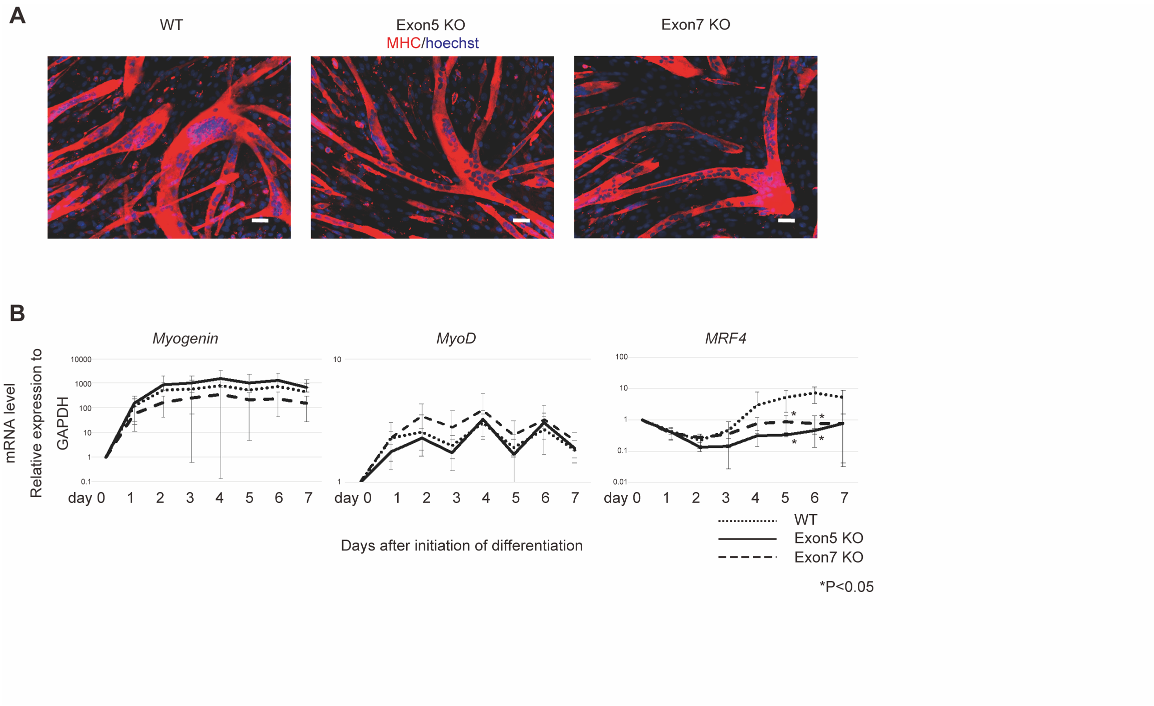

Supplementary Fig. 1. Gene expression during myotube differentiation in C2C12.

A. Representative images of Hoil-1l KO C2C12 myotubes stained by MHC. Bar=50μm
B. MRF4 expressions were significantly decreased in Hoil-1l knockout C2C12 myotubes. (*P<0.05 WT vs Exon 5 and Exon 7 KO.)

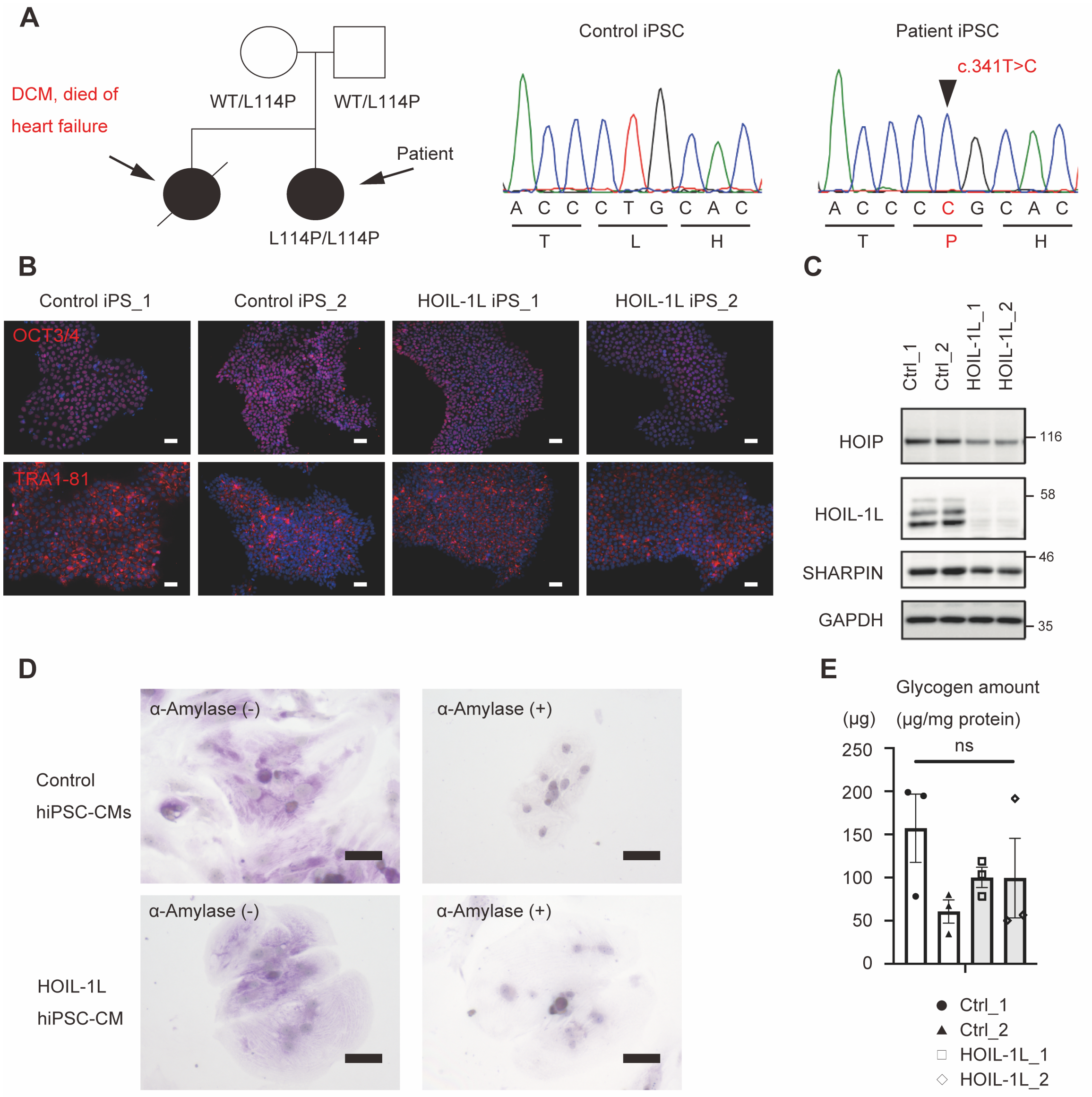
A
DCM, died of
heart failure
WT/L114P
WT/L114P
Patient
L114P/L114P
Control iPSC
A C C C T G C A C
T L H
Patient iPSC
c.341T>C
A C C C C G C A C
T P H
B
Control iPS_1
Control iPS_2
HOIL-1L iPS_1
HOIL-1L iPS_2
OCT3/4
TRA1-81
C
Ctrl_1
Ctrl_2
HOIL-1L_1
HOIL-1L_2
HOIP
HOIL-1L
SHARPIN
GAPDH
116
58
46
35
D
Control
hiPSC-CMs
HOIL-1L
hiPSC-CM
α-Amylase (-)
α-Amylase (+)
E
Glycogen amount
(μg) (μg/mg protein)
ns
250
200
150
100
50
0
Ctrl_1
Ctrl_2
HOIL-1L_1
HOIL-1L_2

Supplementary Fig. 2. Characterization of HOIL-1L deficient patient-specific hiPSC-CMs and amylopectinosis assay in hiPSC-CMs.

A. Schematic pedigrees of a family with HOIL-1L deficiency. The patient is indicated by arrow, and her old sister died of cardiac failure. Her parents have heterozygous L114P missense mutation on exon 4 of the HOIL-1L gene and the patients has homozygous L114P missense mutation on HOIL-1L gene. Two cell lines were established, and L114P (c.341T>C) were confirmed by sequence analysis of PCR-amplified genomic DNA.
B. Immunofluorescence for OCT3/4 and TRA-1-81 of representative hiPSC colonies of controls and the patient.
C. Western blotting analysis of lysates of hiPSC from control, patient specific and HOIL-1L KO.
D. Amylopectinosis, which is not able to be digested by α-amlyase, was not detected both in control and HOIL-1L deficiency hiPSC-CMs. Bar=50μm
E. Glycogen amounts of Control and HOIL-1L deficiency hiPSC-CMs were not significantly different, either.

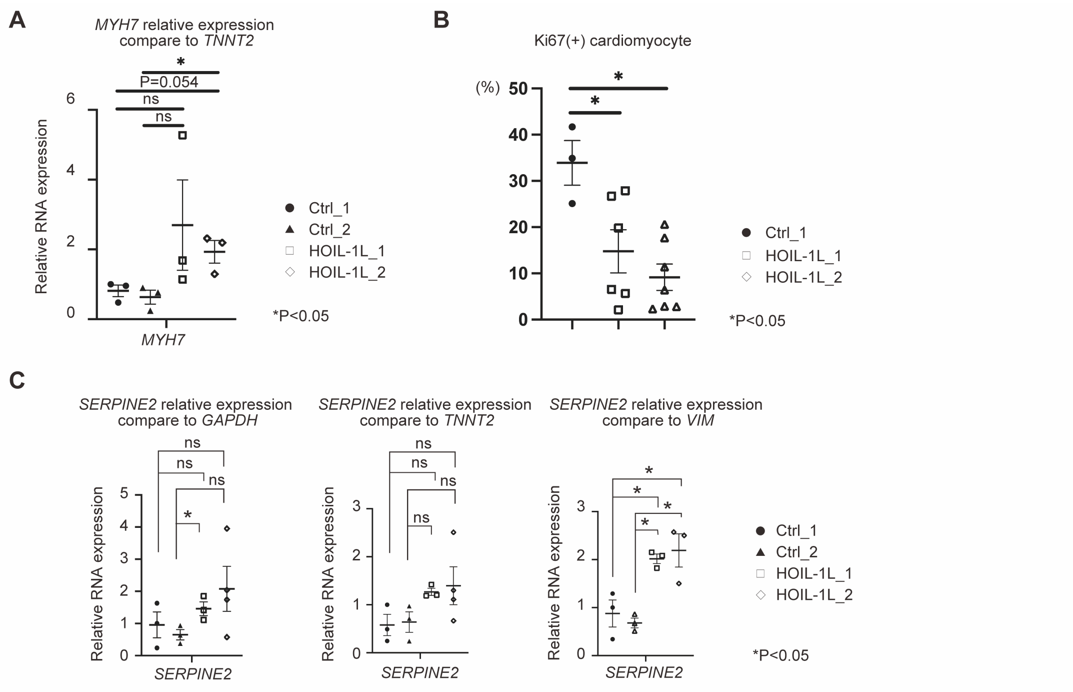

Supplementary Fig. 3. Analysis of *MYH7* and *SERPINE2* by RT-qPCR, and Ki67 positive cardiomyocyte by FACS.

A. RT-qPCR of hiPSC-CMs show higher *MYH7* expression in HOIL-1L deficiency patient hiPSC-CMs than control hiPSCs-CMs.

B. The ratio of Ki67 positive cardiomyocyte is significantly lower in HOIL-1L deficiency patient hiPSC-CMs than control hiPSCs-CMs.

C. RT- qPCR of hiPSC-CMs show *SERPINE2* overexpression in not *GAPDH* and *TNNT2* positive HOIL-1L deficiency patient hiPSC-CMs, but *VIM* positive HOIL-1L deficiency patient hiPSC-cardiac fibroblasts. (N=3-4 independent experiments, *P<0.05)

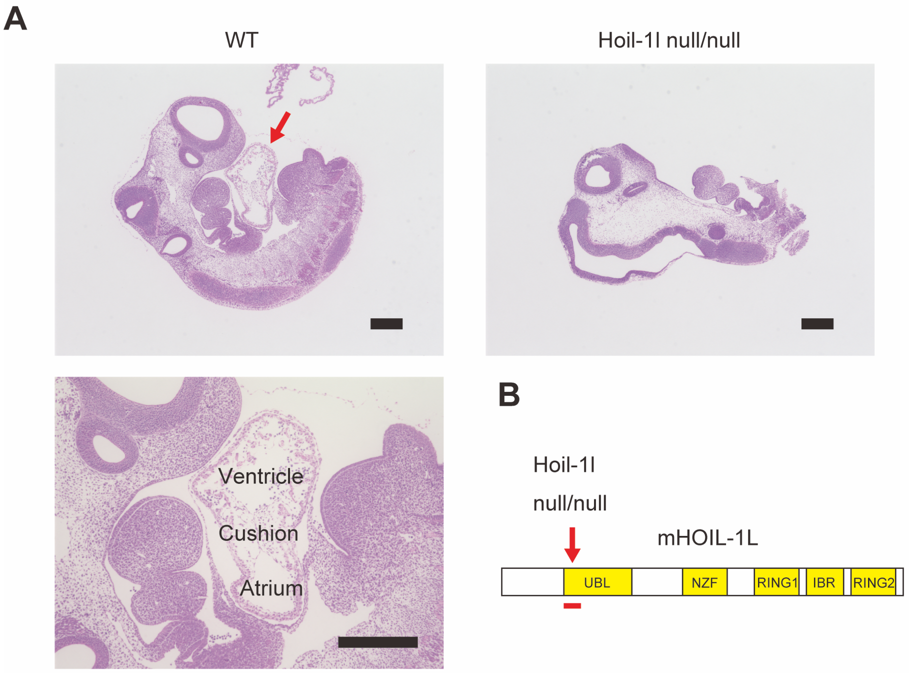

Supplementary Figure 4. Hematoxylin-eosin staining of Hoil-1l KO fetal mouse

A. Representative image of Hematoxylin-eosin staining on E10.5 whole embryo paraffin embedded sections. A wild type mouse embryo heat developed normally (red arrow of left upper panel, magnified image is presented in left lower panel). Hoil-1l null/null mouse shows completely defect of cardiac development accompanies with embryonic lethality (right upper panel). Bar=300μm
B. Schematic representation of Hoil-1l null/null mutation from Fujita et al (2018). *Cell Report 23, 1192-1204*

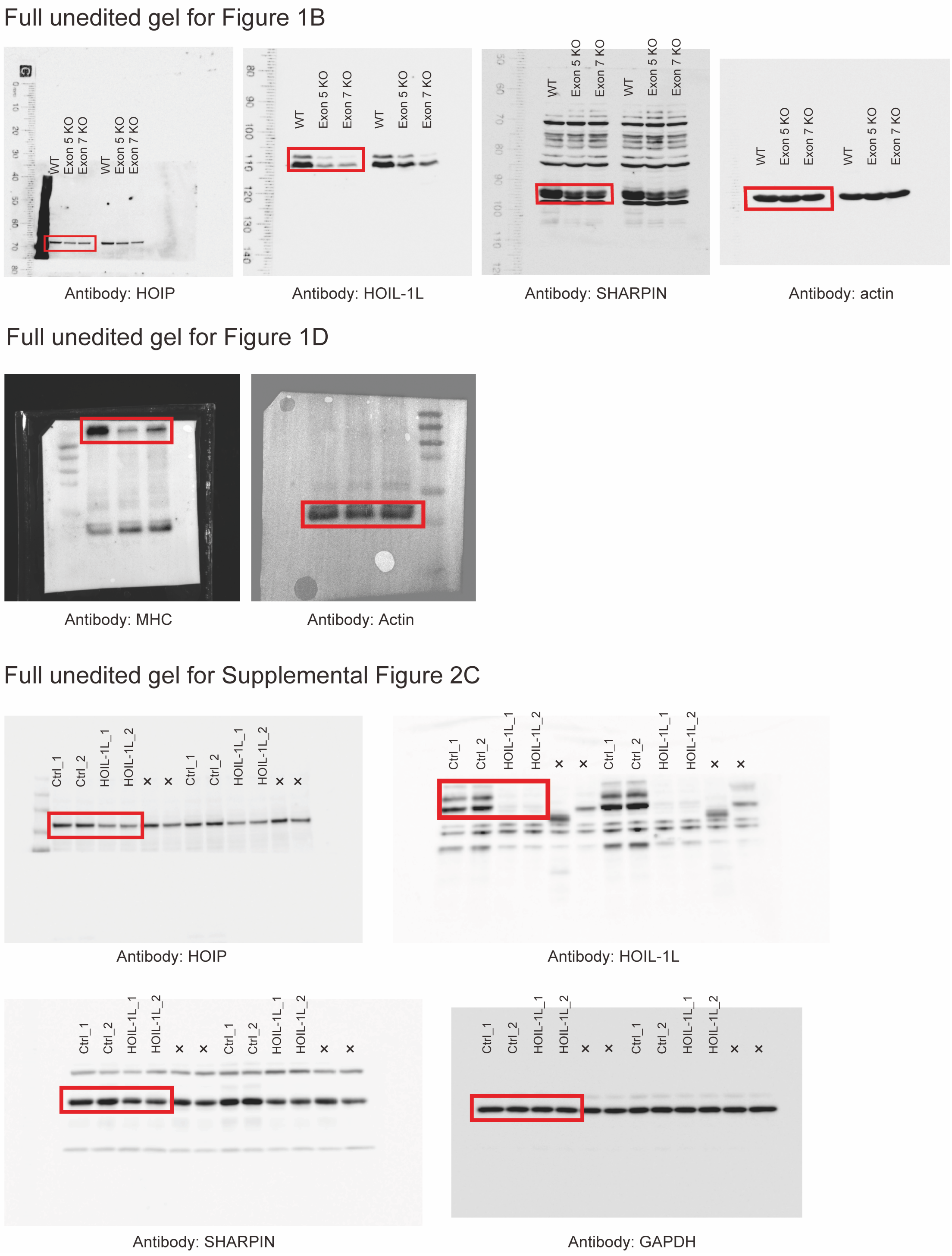

Supplementary Figure 5. Unedited blots and gel imaging for each figure.
